# Statistical Methods to Study Timing of Vulnerability with Sparsely Sampled Data on Environmental Toxicants

**DOI:** 10.1289/ehp.1002453

**Published:** 2010-12-08

**Authors:** Brisa Ney Sánchez, Howard Hu, Heather J. Litman, Martha Maria Téllez-Rojo

**Affiliations:** 1 Department of Biostatistics and; 2 Department of Environmental Health Sciences, University of Michigan School of Public Health, Ann Arbor, Michigan, USA; 3 New England Research Institutes, Watertown, Massachusetts, USA; 4 Division of Statistics, Center for Evaluation Research and Surveys, National Institute of Public Health, Cuernavaca, Morelos, Mexico

**Keywords:** exposure patterns, lead, multiple informants, neurodevelopment, sensitive periods of development

## Abstract

**Background:**

Identifying windows of vulnerability to environmental toxicants is an important area in children’s health research.

**Objective:**

We compared and contrasted statistical approaches that may help identify windows of vulnerability by formally testing differences in exposure effects across time of exposure, incorporating continuous time metrics for timing of exposure, and efficiently incorporating incomplete cases.

**Methods:**

We considered four methods: 1) window-specific and simultaneously adjusted regression; 2) multiple informant models; 3) using features of individual exposure patterns to predict outcomes; and 4) models of population exposure patterns depending on the outcome. We illustrate them using a study of prenatal vulnerability to lead in relation to Bayley’s Mental Development Index at 24 months of age (MDI24).

**Results:**

The estimated change in MDI24 score with a 1-log_e_-unit increase in blood lead during the first trimester was −2.74 [95% confidence interval (CI), −5.78 to 0.29] based on a window-specific regression. The corresponding change in MDI24 was −4.13 (95% CI, −7.54 to −0.72) based on a multiple informant model; estimated effects were similar across trimesters (*p* = 0.23). Results from method 3 suggested that blood lead levels in early pregnancy were significantly associated with reduced MDI24, but decreasing blood leads over the course of pregnancy were not. Method 4 results indicated that blood lead levels before 17 weeks of gestation were lower among children with MDI24 scores in the 90th versus the 10th percentile (*p* = 0.08).

**Conclusions:**

Method 2 is preferred over method 1 because it enables formal testing of differences in effects across *a priori*–defined windows (e.g., trimesters of pregnancy). Methods 3 and 4 are preferred over method 2 when there is large variability in the timing of exposure assessments among participants. Methods 3 and 4 yielded smaller *p*-values for tests of the hypothesis that not only level but also timing of lead exposure are relevant predictors of MDI24; systematic power comparisons are warranted.

Identifying critical windows of vulnerability to environmental toxicants is an important area in children’s health research. Critical windows of vulnerability are defined as periods during life when an exposure causes a stronger deficit in health later in life compared with other periods when exposure (could have) occurred (Barr et al. 2000; Selevan et al. 2000; West 2002). The research question is often formulated as “Is the exposure more strongly associated with the health outcome if it occurred in time window 1, 2, …, or *n*?” Formulating the question in terms of discrete time windows is advantageous from a clinical and practical perspective. However, considering timing of exposure in a continuous fashion may be more advantageous from an analytical standpoint.

We discuss three statistical approaches that may be useful in studies of windows, or timing, of vulnerability; two of these use time of exposure as a continuous variable. We compare the proposed methods with commonly used statistical approaches such as fitting separate regression models for each potential window and fitting simultaneously adjusted multiple regressions that include all exposure measures (time windows) in one model.

As exposition, we use data from a study of lead exposure during pregnancy and mental development. We refer readers to published studies of lead exposure for information regarding the toxicologic effects of lead on development and the relevance of time windows of vulnerability in that context (Hu et al. 2006; Schnaas et al. 2006). We focus on statistical modeling issues and apply all methods to the same data so that the inferences and interpretations across methods can be compared.

## Materials and Methods

### Data source

We use data from an ELEMENT (Early Life Exposures in Mexico to Environmental Toxicants) study cohort of mother–child pairs recruited during pregnancy or before conception (Téllez-Rojo et al. 2004). We use maternal blood lead concentrations ascertained during study visits scheduled within each trimester. Low birth weight (< 2,500 g) and preterm (< 37 weeks) children were excluded. Children’s mental development was measured at 24 months using Bayley’s Mental Development Index (MDI24) (Bayley 1993). We also collected other participant characteristics (Table 1); data collection procedures are reported elsewhere (Hu et al. 2006; Téllez-Rojo et al. 2004). This analysis was restricted to *n* = 169 participants with complete covariates (mother’s age and IQ, breast-feeding duration, and child’s sex, height, weight, and blood lead level at 24 months) and at least one measure of prenatal lead exposure.

Participants gave written informed consent before data collection. The study was approved by the institutional review boards of the hospitals where participants were recruited, the National Institute of Public Health of Mexico, Brigham and Women’s Hospital, Harvard School of Public Health, and the University of Michigan.

### Notation

For each subject *i*, assume *X_1i_*, *X_2i_*, *… X_Ki_* are measures of exposure taken during *K_i_* time points *t_1i_, t_2i_*, *…, t_Ki_*; *Y_i_* is the health outcome, and *Z_i_* are mean-centered covariates. For the lead study, *Y_i_* is MDI24, *X_1i_*, *X_2i_*, *… X_Ki_* are log_e_(maternal blood lead levels, micrograms per deciliter), and *Z_i_* are maternal age, maternal IQ, child’s sex, and child’s 24-month weight and height-for-age *z*-score (Kuczmarski et al. 2002). For methods 1 and 2, we assume the *K_i_* measures of exposure fall within *K* time windows (i.e., *K* = three trimesters).

### Method 1: separate and simultaneously adjusted multiple linear regression models

A commonly used statistical approach in studies of prenatal windows of vulnerability is to fit regression models for each potential window (Aguilera et al. 2009; Bell et al. 2007; Hu et al. 2006; Meyer et al. 2007; Mohorovic 2004; Parker et al. 2005). The magnitude and significance of the regression coefficients are compared to draw conclusions about which window of exposure may be more important.

This approach is intuitive, but it has two assumptions that are not always discussed. First, the exposure is assumed constant within the window. For example, a single maternal blood lead measure during the first trimester is assumed to be representative of average exposure during the entire trimester. Second, although it is recognized that “the closer we look, the more evident it is that often there is not a uniform response within a given window” (Barr et al. 2000), the exposure effect (e.g., the effect of prenatal lead exposure on mental development) is assumed to be constant within a time window. When these assumptions are violated, the estimated effects within a predefined window may be biased toward or away from the null, depending on the true nature of the exposure effect and the time at which exposures are measured. Specific examples are shown in the Supplemental Material, Figures 1 and 2 (doi:10.1289/ehp.1002453).

The approach is also subject to other statistical limitations. First, estimating separate regressions for each time window precludes formal testing of the differences in effects across time windows. Interpreting nonoverlapping confidence intervals (CIs) as proof that effects differ is not valid, because the estimated coefficients are not statistically independent. This issue is analogous to the incorrect choice of a two-sample *t*-test over a (correct) paired *t*-test for testing differences between repeated measures within an individual. Second, it is possible that not all regressions will be based on the same group of subjects because of missing data. For example, a popular approach to maximize the sample size for each regression is to include all observations with data for a given time window in the regression for that time window, even though some may be excluded from models of other time windows because of missing data. However, the available case approach is valid only if data are missing completely at random (MCAR) (Little and Rubin 2002), and it is not possible to verify this assumption based on available (nonmissing) data (for example, not finding significant differences in bivariate analyses comparing subjects with complete versus incomplete data is not sufficient proof of MCAR). Third, because the same outcome is used in all regressions, the hypotheses tested are not independent, and precision may be over- or underestimated because the correlations among the residual errors from the regressions are ignored.

A straightforward alternative to fitting separate regressions for each time window is to fit a single simultaneously adjusted regression model that includes all time windows. An advantage of this approach is that estimates of the independent effect of exposure during each window may be obtained (Ha et al. 2007; Lubin et al. 1997). However, the approach is not always feasible because of collinearity issues (e.g., inflated standard errors, unstable regression coefficients). Further, models are restricted to observations that have complete data for all variables.

We estimated separate and simultaneously adjusted multiple linear regression models to estimate trimester-specific associations between MDI24 and log_e_-transformed maternal blood lead levels.

### Method 2: multiple informants

Multiple informant data refers to information gathered from different individuals or sources used to measure the same construct (Horton et al. 1999; Litman et al. 2007b). One classic example is where mother and child both respond to questions regarding the physical activity of the child, so that the mother and child are the informants. Methods for multiple informant data also can be applied when exposure information is obtained for the same individual at different time points (windows) by treating the exposure windows as informants. Multiple informant methods can be used to test whether the information relayed by different informants (in this case, whether exposure is measured during different time windows) relates in the same manner to an outcome of interest. Although this approach retains the interpretation (and assumptions) of a set of separate multiple regressions (by providing a single estimate of effect for exposure in each time window), it also provides a way to test differences in associations between the exposure and the outcome across time windows.

#### Model

The associations of primary interest are estimated by β*_1k_* from window-specific multiple regressions *Y_i_* *=* β*_0k_* *+* β*_1k_X_ki_* *+* β*_2k_Z_i_* *+* ɛ*_ki_*, for window *k = 1*,*2*, *…, K*. The multiple informants approach jointly estimates the regression models.

Joint estimation enables us to impose and test constraints on the regression coefficients across exposure windows. That is, test whether the exposure coefficients are equal across time windows, *H_o_*: β*_11_* *=* β*_12_* *= … =* β*_1K_* versus *H_a_*: at least one β*_1K_* differs. Parameter estimation can be conducted using generalized estimating equations (GEE) or maximum likelihood (ML) estimation (Horton et al. 1999; Litman et al. 2007a; Pepe et al. 1999). Details regarding data structure, model fitting, hypothesis testing, and macros to analyze the data using this method are provided in the Supplemental Material (doi:10.1289/ehp.1002453).

ML estimation proceeds by modeling the exposure at different windows and the outcome as having a *K* + 1 multivariate normal distribution (i.e., exposure is considered a random variable) and subsequently obtaining the distribution of the outcome conditional on the exposure. The test of equal association between exposure and outcome across time windows can also be performed on standardized regression coefficients (i.e., adjusted correlation coefficient denoted ρ*_1k_* = σ*_Xk_*β*_1k_*/σ*_Y_*, *k* = 1, …, *K*, where σ*_Xk_* is the SD of the exposure at window *k*, and σ*_Y_* is the SD of the outcome. Testing adjusted correlation coefficients may be desirable when the variability of the exposure changes over time or if protocols for measuring exposure change across time windows. ML can account for exposure and outcome data missing at random (MAR) (Little and Rubin 2002) and is robust to distributional assumptions when missing data are MCAR (Litman et al. 2007b). MAR means that the fact the data are missing is independent of the actual missing value, after accounting for other observed participant characteristics. MCAR means that the fact the data are missing is independent of the missing value as well as other observed data and hence is more restrictive than MAR.

The GEE approach embeds separate linear regression models for each time window into a unified set of estimating equations. In contrast to the ML approach, the exposures are not considered random (dependent) variables. However, unless more sophisticated methods are used (Horton and Lipsitz 1999), the GEE method retains the MCAR assumption for the missing data.

We applied the multiple informant approach to the ELEMENT study data. For the GEE approach we used a score type test of the hypothesis *H_o_*: β*_11_* *=* β*_12_* *=* β*_13_* [degrees of freedeom (df) = 2]. Because the variance of the exposure during trimester 2 was smaller than during the other two trimesters, in the ML approach we tested for homogeneity of standardized exposure effects across time windows (*H_o_*: ρ*_11_* *=* ρ*_12_* *=* ρ*_13_*) using a likelihood ratio test (df = 2). The *p*-values for these tests are denoted p*_int_*, alluding to an interaction between exposure level and timing of exposure.

#### Method 3: individual patterns of exposure in relation to outcome

When assumptions regarding the timing of exposure in methods 1 and 2 are violated (i.e., when timing of exposure measurement or the effect of exposure on the outcome varies within time windows), when a large number of time windows are considered, or when the number of measurement occasions for the exposure varies across participants, the multiple informant approach may no longer be feasible. An alternative is to model the pattern of exposure for each individual over time and then relate exposure features to the outcome.

##### Model

The underlying idea in this approach is to reduce the number of exposure measures for each individual from *K_i_*, which may vary across participants, to a smaller number equal across participants. A simple example where the exposures are summarized into two exposure features is to model the exposures *X_ik_* using a random intercepts and random slopes model, *X_ik_* *=* θ*_0i_* *+* θ*_1i_* *t_ik_* *+* ɛ*_ik_*, where *k =* 1*, …, K_i_* represents the *k^th^* measurement occasion. The random effects θ*_0i_*, and θ*_1i_* are person-specific intercepts and slopes that jointly describe the pattern of exposure for individual *i* and have a population mean θ *=* (θ*_0_,*θ*_1_*) and variance Φ. Next, the outcome is modeled in relation to the exposure features θ*_0i_*, and θ*_1i_*, for example, *Y_i_* *=* β*_0_* *+* β*_11_* θ*_0i_* *+* β*_12_* θ*_1i_* *+* β*_2_* *Z_i_* *+* ɛ*_i_*. [Details regarding data structure, model fitting, hypothesis testing, and macros to analyze the data using this method are provided in the Supplemental Material (doi:10.1289/ehp.1002453).]

For the ELEMENT study, where a few measures of exposure were available, we employed a model where a random intercept and slope were the only subject-specific exposure features. The time variable was centered at 7 weeks, the middle of the first trimester, such that θ*_0i_* represents blood lead levels during gestational week 7. First, the exposure was modeled as *X_ik_* *=* θ*_0i_* *+* θ*_1i_* (*t_ik_* −*7*) *+* ɛ*_ik_*, with *t_ik_* being gestational time in weeks. Because week 7 is the midpoint of the first trimester, θ*_0i_* can also be interpreted as the average exposure for individual *i* during the first trimester, so that study participants with a random intercept θ*_0i_* higher than the mean θ*_0_* would have a higher than average lead exposure in early pregnancy. The slope θ*_1i_* represents the subject’s rate of change in exposure across the pregnancy. For example, if the population slope θ*_1_* was negative (indicating declining lead levels across pregnancy), a study participant with a random slope θ*_1i_* larger than the mean θ*_1_* would have a slower than average rate of decline in lead levels over the course of pregnancy. We modeled the outcome (MDI24) as *Y_i_* *=* β*_0_* *+* β*_11_* θ*_0i_* *+* β*_12_* θ*_1i_* *+* β*_2_* *Z_i_+* ɛ*_i_*, where β*_11_* is interpreted as the association between exposure during the first trimester and MDI24, and β*_12_* is the association between changes in exposure over the course of pregnancy and MDI24.

We fit the model for the exposures and the outcome model jointly using ML (Wang et al. 2000), which yields consistent and efficient estimates of model parameters. Heuristically, a two-step approach also could be used where empirical Bayes estimates of the exposure features are obtained, for example, θ̂*_0i_*, and θ̂*_1i_*, and then substituted in the outcome model. However, the two-step approach gives inconsistent (likely attenuated) estimates of the regression coefficients (Wang et al. 2000).

Method 3 does not require that participants be measured at the same time points, whereas methods 1 and 2 assume the timing of measurements is similar across participants. Furthermore, because the timing of exposure is not discretized into windows, this approach is more useful than the multiple informant approach when windows are not well defined or when little prior information about the etiologically relevant windows are is available. It requires that each participant has sufficient information such that the features can be reliably estimated (i.e., at least one more measurement than features in most participants). In the lead example, at least three measurements are required for most participants, because two features are estimated. Although the interpretation of model parameters may not be as straightforward because the reference to discrete windows is lost, conclusions about the relative importance of broad time periods can still be drawn. In the lead example, this method helps answer the question “After accounting for first-trimester exposure, does changing the exposure in subsequent trimesters matter?” In the lead example, this approach assumes a constant rate of decline in exposure, and the association of exposure with outcome over the course of pregnancy diminishes (or increases) at a linear rate.

#### Method 4: population pattern of exposure given the outcome

The fourth method consists of describing the population-average exposure pattern for levels of the outcome. For example, in ELEMENT, this method can be used to compare the pattern of exposure for children who achieve high MDI24 scores with the pattern in children with low scores. This approach differs from methods 1–3 because it models the exposure given the health outcome.

##### Model

The exposure is modeled as *X_ik_* = *f*_0_(*t_ik_*) + *f_1_*(*t_ik_*)*Y^c^_i_* + δ*_ik_*, where *Y^c^_i_* is the outcome for subject *i* centered at the sample mean (*Y^c^_i_* = *Y_i_* − *Ȳ*) or centered at its predicted value (*Y^c^_i_* = *Y_i_* − *Ŷ_i_*, where *Ŷ_i_* is the predicted outcome given *Z_i_*) given factors other than exposure (e.g., suspected confounders and possibly other independent predictors of the outcome). The residuals δ*_ik_* are assumed to have mean zero and covariance Δ within individuals but are independent across individuals. The term *f_0_*(*t_ik_*) represents the exposure pattern over time for those with an average outcome, which can be modeled as a parametric function [e.g., *f_0_*(*t_ik_*) = α*_00_* + α*_01_t_ik_* + α*_02_t*^2^*_ik_*] or a semiparametric curve (e.g., penalized splines). The term *f_0_*(*t_ik_*)*Y^c^_i_* quantifies the differences in exposure over time across levels of the outcome. Both *f_1_*(*t_ik_*) and *f_0_*(*t_ik_*) are modeled in the same fashion [e.g., *f_1_*(*t_ik_*) = α*_10_* + α*_11_t_ik_* + α*_12_t*^2^*_ik_* if *f_0_*(*t_ik_*) is modeled as a quadratic function]. The coefficients α*_10_*, α*_11_*, α*_12_* jointly describe the pattern of exposure curve over time.

An overall test of association between exposure and outcome involves testing *H_o_*: *f_1_*(*t*) *= 0* vs. *H_a_*: *f_1_*(*t*) *≠ 0*. When *f_1_*(*t*) is given by a quadratic function, then the null hypothesis is *H_o_*: α*_10_* = α*_11_* = α*_12_* = 0 versus *H_a_*: at least one α differs from zero. Testing whether the exposure pattern over time is associated with outcome amounts to testing *H_o_*: α*_11_* = α*_12_* = 0 versus *H_a_*: at least one α differs from zero. For timing of susceptibility (i.e., whether exposure effects vary depending upon the timing of exposure), the more relevant hypothesis is the latter.

The estimation of this model can be conducted in two steps. First, a model for the outcome is estimated where only covariates *Z_i_* are predictors. The residuals *Y^c^_i_* = *Y_i_* − *Ŷ_i_* are then constructed, and the model for *X_ik_* (i.e., a model of the exposure conditional on the outcome) is estimated. Details regarding data structure, model fitting, hypothesis testing, and macros to analyze the data using method 4 are provided in the Supplemental Material (doi:10.1289/ehp.1002453).

In the ELEMENT data, we obtained *Y^c^_i_* = *Y_i_* − *Ŷ_i_*, and interpreted it as the deviation of the individual from the expected MDI24 score given mother’s age, mother’s IQ, duration of breastfeeding, sex, and weight and height z-score at 24 months. For example, *Y^c^_i_* will be positive when the MDI24 score of a child is higher than predicted given the characteristics listed above. We then estimated the exposure model using *t_ik_* as gestational time in weeks and nonparametric and quadratic models for *f_0_*(*t*) and *f_1_*(*t*). Finally, we constructed the predicted exposure pattern (back-transformed to natural units of blood lead) for children in the 10th and 90th percentile of the covariate-adjusted MDI distribution and determined the relative difference in exposure between the 10th and 90th percentiles of the covariate-adjusted MDI distribution across time by exponentiating the difference in the predictions of log_e_-transformed blood lead.

## Results

The study visits occurred, on average, toward the end of each trimester, with considerable variability in their timing (Table 1). Notably, although the earliest visit among all study participants was at 3.8 weeks of gestation, the average first-trimester visit occurred at 13.7 weeks. The variability in timing of measurements raises concerns about interpretation of regression coefficients as the association between exposure during each trimester and mental development and was the primary motivation for seeking alternative analytical methods for this type of data. Nevertheless, we keep the language of trimesters in the subsequent descriptions of the results for methods 1 and 2.

The estimated associations from the separate and simultaneously adjusted regression models were as follows: −5.42 (95% CI, −10.2 to −0.64) and −2.74 (95% CI, −5.78 to 0.29) MDI24 points per 1-log_e_-unit increase in blood lead in the first trimester, respectively (Table 2). Although not significant, the coefficients for trimesters 2 and 3 from the simultaneously adjusted regression were positive (suggesting higher MDI24 scores with higher lead exposure) and imprecise, which would be consistent with collinearity. The coefficients from separate regressions were all negative and decreased in strength across trimesters, with the strongest association observed at trimester 1. However, none of the estimated effects were statistically significant (*p* > 0.05).

The estimated associations between first-trimester exposure and MDI24 from the multiple informant approach were −2.74 (95% CI, −5.82 to 0.33) and −4.13 (95% CI, −7.54 to −0.72) MDI24 points per 1-log_e_-unit increase in blood lead from GEE and ML estimation, respectively (Table 2). As is always the case (Litman et al. 2007a), the GEE estimates were equal to the point estimates obtained from separate regressions, but the CIs varied slightly because the GEE method takes into account within-individual correlation across the time windows. Because there were missing data, the ML and GEE approaches do not give the same estimates. The ML approach estimated a stronger association for trimester 1 (e.g., β_GEE_ = −2.74 vs. β_ML_ = −4.13). Although a trend of increasing effect of lead in earlier times during pregnancy is suggested, tests of a varying exposure effect were not significant (GEE *p_int_* = 0.56, score test of equal regression coefficients; MLE *p_int_* = 0.23, likelihood ratio test of equal standardized coefficients). The ML approach detected a significant association at trimester 1 and exhibited a smaller *p*-value than GEE for the test of differences in association across trimesters. This reflects greater efficiency of ML estimation relative to GEE when data are missing and when differences in standardized regression coefficients are tested, in addition to using score versus likelihood ratio tests.

The exposure model parameters from method 3 indicate that the average exposure at 7 weeks of gestation was 1.90 log_e_(blood lead) units (approximately = 6.69 μg/dL), SD 0.49 (Table 3). This is consistent with the average and SDs of the observed measures during trimester 1 (Table 1). There was a significant average decline across pregnancy (average linear decline is 0.04 ln(blood lead) units per 12 weeks) (Table 3).

The regression coefficients for blood lead level at 7 weeks and the average change in blood lead level are expressed in MDI points associated with a 1-SD change in these predictors. A 1-SD increase in log_e_-transformed blood lead levels at 7 weeks was significantly associated with a 2.11-point decrease in MDI24 (95% CI, −0.01 to 4.23). Although the changes in exposure over the course of pregnancy were not significantly associated with MDI24 (*p* = 0.73), the positive association, indicating that a slower rate of decline in blood lead (i.e., sustained exposure) was associated with a 0.58 point increase in MDI24, was unexpected. However, this relation may have resulted from collinearity; although the random intercept and slope were less correlated than the raw lead concentrations (e.g., correlation between trimester 1 and 2 measurements is 0.66), they were still significantly and negatively correlated (correlation is −0.56).

Notably, the correlation between the predicted random intercept and the observed blood lead level at trimester 1 was 0.97, indicating that the random intercept was closely related to the measured exposure at the first visit. The advantage of the random intercept is that it is interpreted as exposure level at 7 weeks for all study participants and can be estimated for participants who did not have a measured blood lead level at trimester 1. This advantage translates to stronger standardized estimates for the association between exposure and MDI [−2.11/0.49 = −4.31 points (95% CI, −8.66 to 0.02) for a 1-log-unit increase in exposure] compared with corresponding estimates based on methods 1 and 2 (2.74 points via separate regressions or GEE, −4.13 points via MLE).

**Figure 1:**
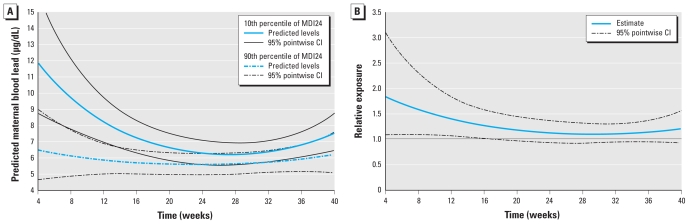
(*A*) Maternal blood lead pattern across gestational period (weeks) for children in the 10th percentile (solid) and 90th percentile (dashed) of the covariate-adjusted MDI24 distribution with 95% pointwise CIs. (*B*) Relative exposure comparing those in the 10th percentile of the MDI distribution with those in the 90th percentile, with 95% pointwise CIs.

The exposure patterns from method 4 were estimated with quadratic functions (the shapes of *f_0_*(*t*) and *f_1_*(*t*) did not deviate significantly from quadratic); the coefficients for these functions are given in Table 4. Lead exposure and MDI24 were significantly associated (*H_o_*: α*_10_* = α*_11_* = α*_12_* = 0, *p* = 0.03) based on this model. The exposure patterns over time comparing high with low achievers marginally differed in their shape over time (*H_o_*: α*_11_* = α*_12_* = 0, *p* = 0.086). Figure 1 portrays the prenatal exposure patterns for children in the 90th and 10th percentile of the covariate-adjusted MDI distribution (top) and their relative difference (bottom). The earliest time at which we estimated exposures was at 4 weeks, the first observed time point among all mothers. As can be observed, the exposure patterns (i.e., the shapes of the curves over time) were similar toward the end of pregnancy but had less overlap earlier in pregnancy. More specifically, children in the 10th percentile of the distribution had significantly higher exposures during the first 17 weeks of pregnancy compared with children in the lower 90th percentile of the distribution. At 7 weeks of pregnancy, those in the 10th percentile had exposure 1.63 (95% CI, 1.09 to 2.45) times higher compared with those in the 90th percentile. The significant relative exposure difference persisted until approximately 17 weeks (relative difference = 1.24; 95% CI, 1.00–1.53).

## Conclusions

Identifying critical windows of vulnerability is an emerging field in children’s health research. Existing statistical approaches used in this area, primarily based on multiple regression, have important limitations: Formal tests for the difference across windows cannot be performed, missing data cannot be easily incorporated, and variation in the timing of exposure cannot be easily accommodated. We presented three alternative approaches that mitigate these limitations. Table 5 summarizes their assumptions, and compares them with multiple regression. To our knowledge, this is the first application of a multiple informants approach (method 2) and population exposure pattern approach (method 4) to studies of timing of vulnerability in environmental epidemiology.

Modifications of multiple regression have been discussed previously in the literature (e.g., Bell et al. 2007; Hornung et al. 2009; Slama et al. 2007, 2008a). These methods involve *a*) including as predictors the residuals from models of the exposure during each window regressed on a reference window; *b*) using ratios of exposure during each window to a reference window; and *c*) including exposures outside the windows of interests (e.g., postnatal exposure) as a control variable (Slama et al. 2007). However, these methods are limited primarily by their lack of ability to incorporate missing data.

Methods beyond multiple regression that address windows of vulnerability have been discussed in the literature under the rubric of exposure–time response models. These models focus on estimating a weight function, *w*(*t*), that measures the relative effect of an exposure increment at time *t* compared with other times (Hauptmann et al. 2000). The time metric *t* can be time at exposure as in our case and other examples in cancer research (Richardson and Wing 1998) or time since exposure, as in studies quantifying latency (Berhane et al. 2008; Langholz et al. 1999; Richardson 2009). These methods, and the ones we propose, are geared toward settings of protracted exposures. Specifically, our method 3 can be seen as a special case of exposure–time response models, because the coefficients corresponding to features θ*_0i_* (level at week 7) and θ*_1i_* (rate of change through pregnancy) can be transformed to obtain an estimate of *w*(*t*) (James 2002). The estimated *w*(*t*) in our case would be a linear function [see Supplemental Material, Figure 4 (doi:10.1289/ehp.1002453)]. Furthermore, related approaches have also been discussed in life course epidemiology (Hallqvist et al. 2004), where one of the approaches is to develop a taxonomy of exposure patterns based on dichotomized exposures at each exposure window and relate the patterns to health outcomes. All of these methods are related, but a distinguishing factor is the time scale on which they focus and whether time of exposure is discretized or treated as continuous.

Although the proposed approaches circumvent some drawbacks of fitting multiple regression models, they are not without limitations. For example, the tests offered by method 2 are low-powered. In principle, method 3 allowed us to flexibly model individual exposure patterns over time; however, relating outcome to many exposure features may become cumbersome to interpret (e.g., individual-level quadratic trends). Method 4 could be improved by incorporating confidence bands instead of pointwise CIs when presenting the results graphically. However, the test of whether population exposure patterns vary over time between outcome groups is valid.

When the windows of vulnerability refer to prenatal windows, inaccurate ascertainment of gestational age may limit the applicability/interpretation of the above methods. Inaccurate measurement of gestational time may arise from incorrect reporting of last menstrual period, for example. This introduces measurement error and may attenuate effect estimates. When gestational time (e.g., weeks) is used to define windows of vulnerability (e.g., trimesters), then even a few days’ error in gestational age could lead to misclassifying the exposure as occurring in one trimester versus another. Furthermore, because of field issues (e.g., rescheduled appointments), some participants may be seen during a later trimester but their visit recorded as occurring within the previous time window. Misclassification of the timing of exposure would lead to biased exposure–outcome associations. Method 2 (like method 1) is not robust to misclassification of the timing of exposure. Methods 3 and 4 may be less affected by this issue, however, because the time is treated as continuous, such that the extent of misclassification is of a lesser magnitude. Other issues of how inaccurate ascertainment of gestational age may introduce bias in exposure–birth outcome studies, even when using ultrasound to ascertain gestational age, have been discussed in the literature (Slama et al. 2008b).

We described several approaches to study timing of vulnerability using an example with few exposure windows and few covariates. Extensions of the approach to incorporate more covariate information on exposure models for methods 3 and 4 or using categorical instead of continuous outcomes are discussed in the Supplemental Material (doi:10.1289/ehp.1002453). Sensitivity analyses are also included in the Supplemental Material, where blood lead measured at 24 months is evaluated both as an extra window using method 2 and as a confounder using all methods.

Uncovering windows of vulnerability to environmental pollutants is a complex question that requires sophisticated data analysis tools. Methods that test exposure effect differences across time of exposure should be employed before concluding which window is most important.

Of the methods presented, method 2 is preferred over method 1, because it enables formal testing of differences in effects across *a priori*–defined windows. Methods 3 and 4 are preferred over method 2 when there is large variability in the timing of exposure across participants, as was the case in the example presented.

## Figures and Tables

**Table 1:** Participant characteristics, ELEMENT study data.

Variable	*n*	Mean ± SD	Min, max
Maternal characteristics			

Ln(blood lead) (μg/dL), T1	139	1.90 ± 0.55	0.095, 3.57
Ln(blood lead) (μg/dL), T2	159	1.78 ± 0.45	0.095, 3.01
Ln(Blood lead) (μg/dL), T3	147	1.85 ± 0.53	0.18, 3.64
Age (years)	169	26.7 ± 5.2	18, 42
IQ	169	89 ± 12.8	55, 120

Child			

Sex (percent male)	169	50.9%	
Weight at 24 months (kg)	169	12 ± 1.5	9.40, 19.30
Height *z*-score at 24 months	169	−0.1 ± 0.93	−3.78, 3.22
Ln(blood lead) at 24 months	169	1.39 ± 0.63	−0.22, 3.60
MDI24	169	91.8 ± 11.6	68, 122
Breast-feeding duration (months)	169	6.4 ± 5.7	0, 24

Timing of sample collection (weeks)			

T1	139	13.7 ± 3.4	3.9, 20.4
T2	159	24.5 ± 2.8	18.0, 33.7
T3	147	35.2 ± 1.9	29.0, 39.0

Correlations among ln(blood lead) levels	T1	T2	T3

Ln(blood lead) T1	1		
Ln(blood lead) T2	0.66	1	
Ln(blood lead) T3	0.56	0.61	1
Ln(blood lead) of child at 24 months	0.17	0.24	0.27

Abbreviations: max, maximum; min, minimum; T, trimester.

**Table 2:** Effect of maternal ln(blood lead) on MDI24, estimated from various approaches.

	Multiple regression (method 1)				Multiple informants approach (method 2, *n* = 169)			
	Simultaneous adjustment^a^		Separate regressions^b^		GEE		MLE	
Trimester	β	95% CI	β	95% CI	β	95% CI	β	95% CI
1	−5.42	−10.20 to −0.64	−2.74	−5.78 to 0.29	−2.74	−5.82 to 0.33	−4.13	−7.54 to −0.72
2	0.88	−5.34 to 7.09	−1.37	−4.81 to 2.07	−1.37	−4.79 to 2.05	−2.98	−6.86 to 0.91
3	1.22	−3.65 to 6.08	−1.15	−4.20 to 1.90	−1.15	−4.18 to 1.88	−2.04	−5.11 to 1.04
*p_int_*^c^		NA		NA		0.56^d^		0.23^e^

Abbreviations: MLE, maximum likelihood estimates; NA, not available.

a *n* = 120.

b For trimester 1, *n* = 139; trimester 2, *n* = 159; trimester 3, *n* = 146.

c Test for hypothesis that estimates are equal across trimesters.

d Score test of homogeneity of coefficients.

e Likelihood ratio tests for homogeneity of standardized estimates.

**Table 3:** Parameter estimates for method 3.

Model parameter or predictor	Estimate	SE	*p*-Value
Exposure model parameters			
θ_0_ (average blood lead level, log_e_)	1.90	0.05	< 0.0001
θ_1_ (average rate of change per 12 weeks)^a^	−0.04	0.02	< 0.01
Random intercept SD	0.49		
Random slope SD	0.17		
Correlation of random intercept (θ*_0i_*) and slope (θ*_1i_*)	−0.56		
Residual SD	0.28		

Predictors in outcome model	β	SE	*p*-Value
Blood lead level at week 7 (θ*_0i_*, random intercept)^b^	−2.11	1.08	0.05
Changes in blood lead level (θ*_1i_*, random slope)^a,c^	0.58	1.68	0.73
Maternal Age (per 5 years)	3.04	0.77	< 0.01
Maternal IQ (per 10 points)	0.76	0.64	0.24
Sex of child	−4.98	1.71	< 0.01
Weight at 24 months	−2.13	0.93	0.02
Height *z*-score at 24 months NA	2.82	1.16	0.01
Breast-feeding duration (per 6 months)	−0.63	0.90	0.48

a Average rate of change is negative; hence, increases in the rate represent slower rates of blood lead level decline.

b Change in MDI24 with a 1-SD increase in blood lead at 7 weeks.

c Change in MDI24 with a 1-SD increase in the rate of blood lead level over the course of pregnancy.

**Table 4:** Parameter estimates for method 4.

Predictor or parameter	Estimate	SE	*p*-Value
Outcome model predictors^a^			
Maternal age (5 years)	2.91	0.79	< 0.001
Breast-feeding duration (6 months)	−0.57	0.91	0.53
Maternal IQ (10 points)	0.85	0.66	0.20
Sex of child	−2.10	0.90	0.02
Child’s weight at 24 months	−1.73	0.68	0.01
Child’s height *z*-score at 24 months	2.72	1.16	0.02
Exposure model parameters^b^			
Average exposure pattern, *f_0_*(*t*)			
α*_00_*	3.12	0.49	< 0.0001
α*_01_*	−1.52	0.69	0.03
α*_02_*	0.58	0.32	0.07
Relationship with MDI24, *f_1_*(*t*)^c^			
α*_10_*	−0.13	0.05	0.02
α*_11_*	0.13	0.08	0.08
α*_12_*	−0.05	0.04	0.17

a Estimates indicate changes in MDI24 per unit change in the predictor.

b Estimates characterize pattern of exposure or relationship between MDI and exposure pattern.

c *H_0_*: *f_1_*(*t*) = 0 vs. *f_1_*(*t*) ≠ 0, *p* = 0.011, *H_0_*: *f_1_*(*t*) = constant versus *f_1_*(*t*) ≠ constant, *p* = 0.086.

**Table 5:** Summary of model assumptions.

Assumption	Method 1: simultaneously adjusted regression	Method 1: separate regressions	Method 2: multiple informants	Method 3: individual exposure patterns	Method 4: population exposure patterns
Assumes all participants have the same timing of exposure?	Yes	Yes	Yes	No	No
Assumes predefined windows?	Yes	Yes	Yes	No	No
Assumes homogeneous exposure effect within window?	Yes	Yes	Yes	No	No
Can test difference of estimated exposure effects across time?	No	No	Yes	Yes	Yes
Minimum number of exposure samples needed per participant	One per window	One per window	At least one^a^	At least two^b^	At least one
Missing data assumptions	MCAR	MCAR	MAR (ML) MCAR (GEE)	MAR	MAR
Assumed time spacing between one window and another?	No restrictions^c^	No restrictions^c^	No restrictions^c^	Some restrictions^d^	Some restrictions^d^
Robust to misclassification of exposure timing?	No	No	No	Somewhat	Somewhat
Subject to collinearity problems?	Yes	No	No	Some	No

a Provided some participants have one in each window.

b Provided most participants have more.

c For example, childhood versus adulthood can be compared, even if only two measures (total) are available.

d Very infrequent measurements or those taken very far apart would make the exposure pattern hard to estimate and interpret.

## References

[b1-ehp-119-409] Aguilera I, Guxens M, Garcia-Esteban R, Corbella T, Nieuwenhuijsen MJ, Foradada CM (2009). Association between GIS-based exposure to urban air pollution during pregnancy and birth weight in the INMA Sabadell Cohort. Environ Health Perspect.

[b2-ehp-119-409] Barr M, DeSesso JM, Lau CS, Osmond C, Ozanne SE, Sadler TW (2000). Workshop to identify critical windows of exposure for children’s health: cardiovascular and endocrine work group summary. Environ Health Perspect.

[b3-ehp-119-409] Bayley N (1993). Bayley Scales of Infant Development.

[b4-ehp-119-409] Bell ML, Ebisu K, Belanger K (2007). Ambient air pollution and low birth weight in Connecticut and Massachusetts. Environ Health Perspect.

[b5-ehp-119-409] Berhane K, Hauptmann M, Langholz B (2008). Using tensor product splines in modeling exposure-time-response relationships: application to the Colorado Plateau Uranium Miners cohort. Stat Med.

[b6-ehp-119-409] Ha M, Mabuchi K, Sigurdson AJ, Freedman DM, Linet MS, Doody MM (2007). Smoking cigarettes before first childbirth and risk of breast cancer. Am J Epidemiol.

[b7-ehp-119-409] Hallqvist J, Lynch J, Bartley M, Lang T, Blane D (2004). Can we disentangle life course processes of accumulation, critical period and social mobility? An analysis of disadvantaged socio-economic positions and myocardial infarction in the Stockholm Heart Epidemiology Program. Soc Sci Med.

[b8-ehp-119-409] Hauptmann M, Wellmann J, Lubin JH, Rosenberg PS, Kreienbrock L (2000). Analysis of exposure-time-response relationships using a spline weight function. Biometrics.

[b9-ehp-119-409] Hornung RW, Lanphear BP, Dietrich KN (2009). Age of greatest susceptibility to childhood lead exposure: a new statistical approach. Environ Health Perspect.

[b10-ehp-119-409] Horton NJ, Laird NM, Zahner GE (1999). Use of multiple informant data as a predictor in psychiatric epidemiology. Int J Methods Psychiatr Res.

[b11-ehp-119-409] Horton NJ, Lipsitz SR (1999). Review of software to fit generalized estimating equation regression models. Am Stat.

[b12-ehp-119-409] Hu H, Téllez-Rojo MM, Bellinger D, Smith D, Ettinger AS, Lamadrid-Figueroa H (2006). Fetal lead exposure at each stage of pregnancy as a predictor of infant mental development. Environ Health Perspect.

[b13-ehp-119-409] James GM (2002). Generalized linear models with functional predictors. J Roy Stat Soc B.

[b14-ehp-119-409] Kuczmarski R, Ogden C, Guo S, Grummer-Strawn LM, Flegal KM, Mei Z (2002). 2000 CDC Growth Charts for the United States: Methods and Development.

[b15-ehp-119-409] Langholz B, Thomas D, Xiang A, Stram D (1999). Latency analysis in epidemiologic studies of occupational exposures: application to the Colorado Plateau Uranium Miners Cohort. Am J Ind Med.

[b16-ehp-119-409] Litman HJ, Horton NJ, Hernandez B, Laird NM, Rao CR, Miller JP, Rao DC (2007a). Estimation of marginal regression models with multiple source predictors. Handbook of Statistics.

[b17-ehp-119-409] Litman HJ, Horton NJ, Hernandez B, Laird NM (2007b). Incorporating missingness for estimation of marginal regression models with multiple source predictors. Stat Med.

[b18-ehp-119-409] Little RJA, Rubin DB (2002). Statistical Analysis with Missing Data.

[b19-ehp-119-409] Lubin JH, Tomasek L, Edling C, Hornung RW, Howe G, Kunz E (1997). Estimating lung cancer mortality from residential radon using data for low exposures of miners. Radiat Res.

[b20-ehp-119-409] Meyer U, Yee BK, Feldon J (2007). The neurodevelopmental impact of prenatal infections at different times of pregnancy: the earlier the worse?. Neuroscientist.

[b21-ehp-119-409] Mohorovic L (2004). First two months of pregnancy—critical time for preterm delivery and low birthweight caused by adverse effects of coal combustion toxics. Early Hum Dev.

[b22-ehp-119-409] Parker JD, Woodruff TJ, Basu R, Schoendorf KC (2005). Air pollution and birth weight among term infants in California. Pediatrics.

[b23-ehp-119-409] Pepe MS, Whitaker RC, Seidel K (1999). Estimating and comparing univariate associations with application to the prediction of adult obesity. Stat Med.

[b24-ehp-119-409] Richardson DB (2009). Latency models for analyses of protracted exposures. Epidemiology.

[b25-ehp-119-409] Richardson DB, Wing S (1998). Methods for investigating age differences in the effects of prolonged exposures. Am J Ind Med.

[b26-ehp-119-409] Schnaas L, Rothenberg SJ, Flores MF, Martinez S, Hernandez C, Osorio E (2006). Reduced intellectual development in children with prenatal lead exposure. Environ Health Perspect.

[b27-ehp-119-409] Selevan S, Kimmel CA, Mendola P (2000). Identifying critical windows of exposure for children’s health. Epidemiology.

[b28-ehp-119-409] Slama R, Darrow L, Parker J, Woodruff TJ, Strickland M, Nieuwenhuijsen M (2008a). Meeting report: atmospheric pollution and human reproduction. Environ Health Perspect.

[b29-ehp-119-409] Slama R, Khoshnood B, Kaminski M (2008b). How to control for gestational age in studies involving environmental effects on fetal growth [Letter]. Environ Health Perspect.

[b30-ehp-119-409] Slama R, Morgenstern V, Cyrys J, Zutavern A, Herbarth O, Wichmann HE (2007). Traffic-related atmospheric pollutants levels during pregnancy and offspring’s term birth weight: a study relying on a land-use regression exposure model. Environ Health Perspect.

[b31-ehp-119-409] Téllez-Rojo MM, Hernandez-Avila M, Lamadrid-Figueroa H, Smith D, Hernandez-Cadena L, Mercado A (2004). Impact of bone lead and bone resorption on plasma and whole blood lead levels during pregnancy. Am J Epidemiol.

[b32-ehp-119-409] Wang CY, Wang NS, Wang SJ (2000). Regression analysis when covariates are regression parameters of a random effects model for observed longitudinal measurements. Biometrics.

[b33-ehp-119-409] West LJ (2002). Defining critical windows in the development of the human immune system. Hum Exp Toxicol.

